# Small bowel diaphragm disease with multiple cluster lesions in one segment of the small bowel mimicking an adhesion band: A case report

**DOI:** 10.1097/MD.0000000000035235

**Published:** 2023-11-24

**Authors:** Youseok Jeong, Jae Hun Chung, Dong Won Lim, Si Hak Lee, Sun-Hwi Hwang, Dong Hoon Shin

**Affiliations:** a Division of Gastrointestinal Surgery, Department of Surgery, Pusan National University Yangsan Hospital, Yangsan, Korea; b Department of Surgery, Pusan National University Yangsan Hospital, Yangsan, Korea; c Department of Pathology, School of Medicine, Pusan National University, Pusan National University Yangsan Hospital, Yangsan, Korea; d Research Institute for Convergence of Biomedical Science and Technology, Pusan National University Yangsan Hospital, Yangsan, Korea; e Pusan National University, School of Medicine, Busan, Korea.

**Keywords:** case report, mechanical obstruction, non-steroidal anti-inflammatory drugs, NSAIDs, small bowel diaphragm disease

## Abstract

**Rationale:**

Small bowel diaphragm disease (SBDD) is a rare case, caused by long-term administration of nonsteroidal anti-inflammatory drugs (NSAIDs). The circumferential diaphragm in the lumen of small bowel causing mechanical obstruction is the characteristic finding.

**Patient concerns:**

A 74-year-old male was transferred to Pusan National University Yangsan Hospital (PNUYH) due to abdominal pain lasting for 2 months. He was treated in the local medical center (LMC) with Levin tube insertion and Nil Per Os (NPO) but showed no improvement.

**Diagnosis:**

According to abdomen-pelvis computed tomography (CT) result, small bowel obstruction due to the adhesion band was identified, showing dilatation of the small bowel with abrupt narrowing of the ileum.

**Interventions:**

Laparoscopic exploration was done but failed to find an adhesion band. An investigation of the whole small bowel was done with mini-laparotomy. At the transitional zone, the intraluminal air could not pass so the segmental resection of small bowel including the transitional zone and end-to-end anastomosis was done.

**Outcomes:**

After surgery, every laboratory finding recovered to the normal range in 4 days, but the patient’s ileus lasted for 8 days. The patient’s symptoms were relieved after defecation, he was discharged on postoperative day 10.

**Lessons:**

For patients who show mechanical obstruction without an operation history but with long-term administration of NSAIDs, the clinicians should suspect small bowel diaphragm disease.

## 1. Introduction

Small bowel diaphragm disease (SBDD) is caused by injury from long-term nonsteroidal anti-inflammatory drugs (NSAIDs) administration.^[1]^ A typical feature of SBDD is multiple lesions consisting of circumferential diaphragms causing obstruction of the lumen by a short length (<5 mm), most commonly located in the small intestine.^[2]^ SBDD is still considered a rare disorder, but it has been increasingly diagnosed as one of the complications of NSAID usage. The pathogenesis of SBDD remains unclear as it is based on a multifactorial theory.^[3]^ As SBDD is an obstructive lesion, clinical manifestations of SBDD are associated with obstructive symptoms, abdominal pain, nausea, and vomiting. In addition to obstructive symptoms, diarrhea, iron-deficiency anemia, Gastrointestinal (GI) tract blood loss, and protein-losing enteropathy can also be present.^[1,4,5]^

Wang et al reported that 41.5% of SBDD patients were preoperatively diagnosed using capsule endoscopy (CE) and double-balloon enteroscopy; 52.1% were diagnosed by laparotomy; and 24.7% were diagnosed by computed tomography (CT).^[3]^ Another study found that the most common retrospective CT finding in SBDD patients was strictures in 92% of cases that ranged in length from 5 mm to 10 cm (median length, 1 cm; mean length, 2.8 cm).^[6]^ As SBDD is a rare disease that requires precise medical history-taking and various imaging tests, cases from different medical institutions should be shared to improve diagnosis in future patients. Therefore, we report a case of multiple diaphragms clustered in 1 segment (about 20 cm) of the small bowel.

## 2. Patient information

The patient was a 74-year-old man with a chronic gastric ulcer. He was treated in the outpatient department of our medical institution. Additionally, he had underlying diseases, including hypertension, spinal stenosis, and benign prostate hyperplasia for more than 10 years. As he had a long history of pain from spinal stenosis, he took naproxen 500 mg 1 tab bid and polmacoxib 2 mg 1 cap as pro re nata for pain control. These pain control medications were taken for at least 3 years.

Before visiting the emergency room (ER) of our institution, he received inpatient treatment for 2 months at a local medical clinic (LMC). At the visit to the local clinic, his chief complaint was abdominal pain due to mechanical obstruction. During inpatient treatment at the local clinic, conservative treatment was maintained with the insertion of a Levin tube for decompression and total parenteral nutrition support. However, the patient showed no improvement in symptoms and complained of worsening abdominal pain and distension. Finally, the patient was transferred to the ER at Pusan National University Yangsan Hospital (PNUYH).

## 3. Clinical findings

The patient chief complaint was abdominal pain and distension. His last day of defecation was 7 days before visiting the ER of PNUYH. On physical examination, there was tenderness in the whole abdomen but no rebound tenderness, abdominal rigidity, or guarding. His vital signs were stable: systolic blood pressure, 140 mm Hg; diastolic blood pressure, 100 mm Hg; and pulse rate, 80 to 90 beats/min. No tachypnea was observed.

## 4. Diagnostic assessment

**Figure 1.**
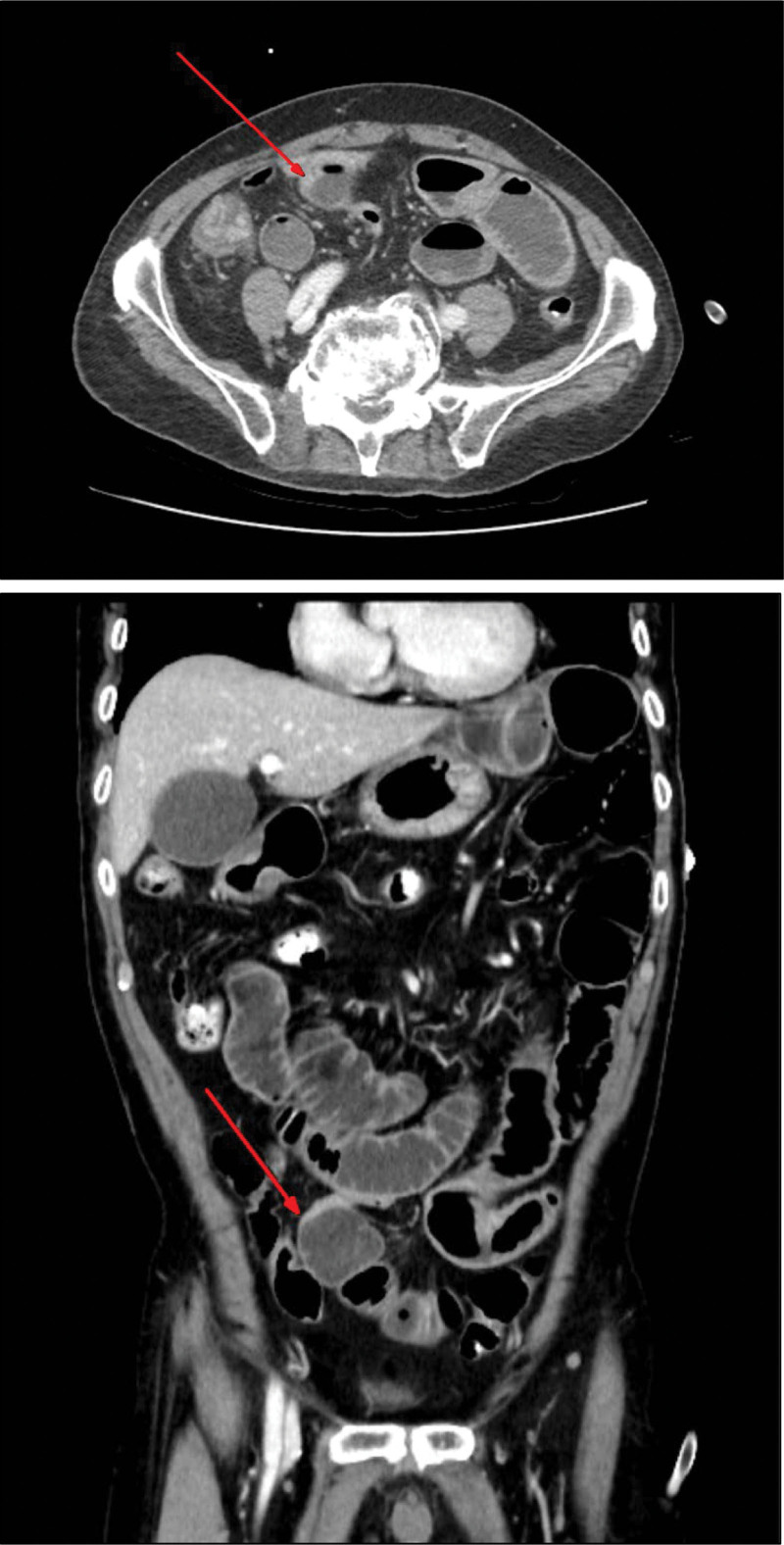
Initial CT abdomen and pelvis. Dilatation of small bowel with abrupt narrowing (red arrow) at the distal ileum suggests simple mechanical obstruction due to the adhesion band.

After arrival at the ER of the PNUYH laboratory, blood tests, erect supine abdominal radiography, and CT of the abdomen and pelvis were performed. Dilatation and edema of the bowel were observed on radiography. Considering the abdominal pelvic CT results, dilatation of the small bowel with abrupt narrowing of the ileum suggested small bowel obstruction due to an adhesion band (Fig. 1). The initial laboratory findings were as follows: white blood cell count, 6370/μL (segmented neutrophil, 60.5%); hemoglobin, 11.7 g/dL; high-sensitivity C-reactive protein, 0.20 mg/dL; and lactic acid, 0.9 mmol/L.

## 5. Therapeutic intervention

**Figure 2.**
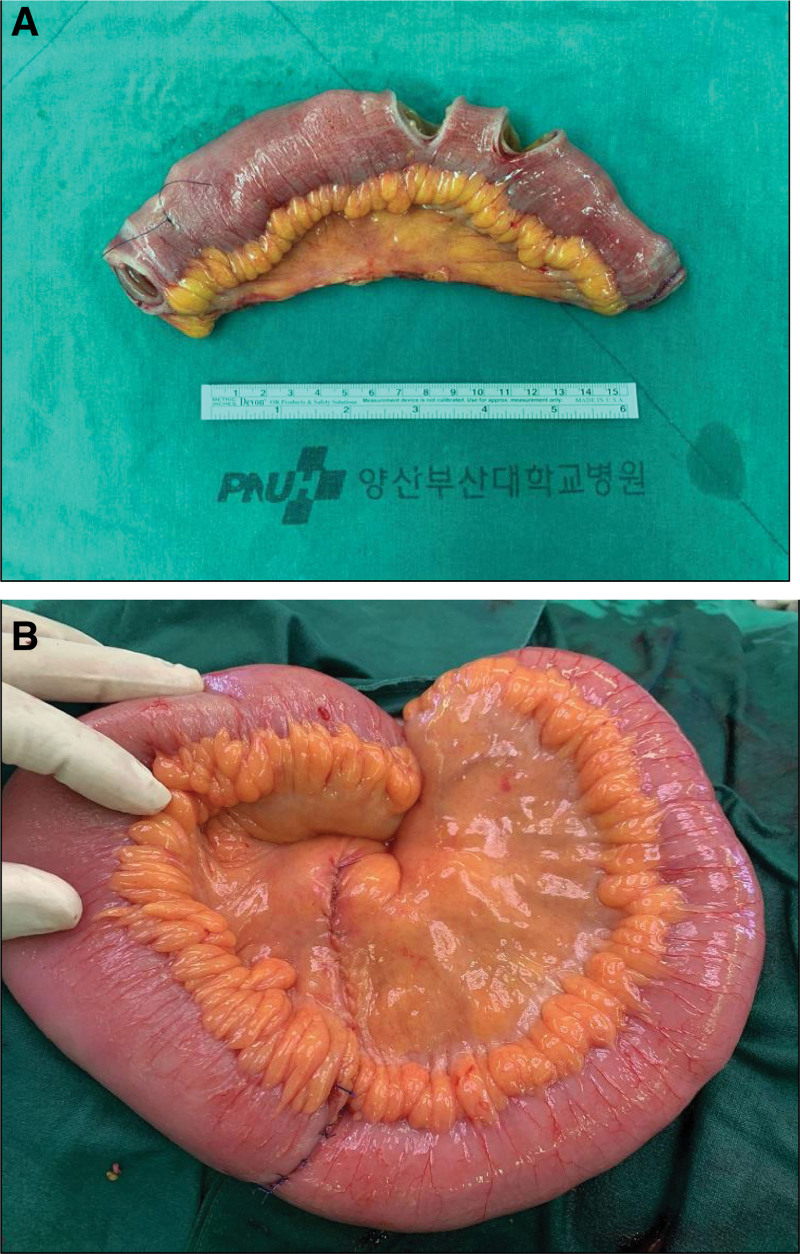
Operative management. (A) The specimen from operation: A segment of resected small bowel. (B) After segmental resection of small bowel: End-to-end anastomosis and mesentery repair was done.

Levin tube insertion and supportive care were maintained for conservative treatment; however, the symptoms were aggravated. After visiting the ER of PNUYH, we had no reason to continue conservative treatment for the patient anymore. Therefore, we performed laparoscopic exploration at the beginning of the emergency surgery. The surgical team failed to find an adhesion band that caused a mechanical obstruction. However, we found a definite transitional zone at the end of the dilated small bowel. The operator decided to convert laparoscopic exploration to mini-laparotomy to investigate the state of the transitional zone and to palpate the entire small bowel. We palpated the whole small bowel from the ligament of Treitz to the ileocecal valve. During palpation of the small bowel, we found that the intraluminal air could not pass through the transitional zone. Segmental resection, including the transitional zone, was performed. The resected segment of the small bowel was approximately 20 cm in length (Fig. 2A). End-to-end anastomosis and mesenteric repair were performed (Fig. 2B).

## 6. Follow-up and outcomes

**Figure 3.**
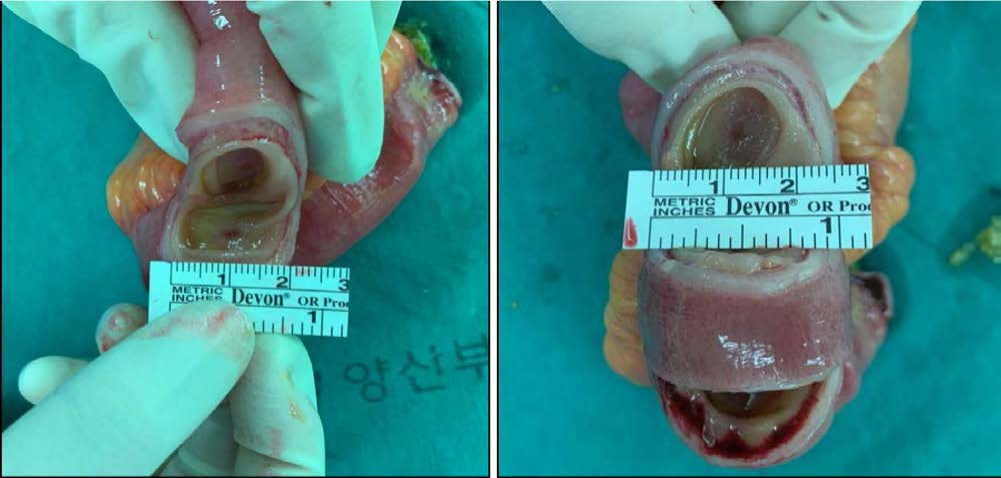
Gross findings in operation room. At least 3 diaphragm like intraluminal obstruction lesion were found in gross inspection.

**Figure 4.**
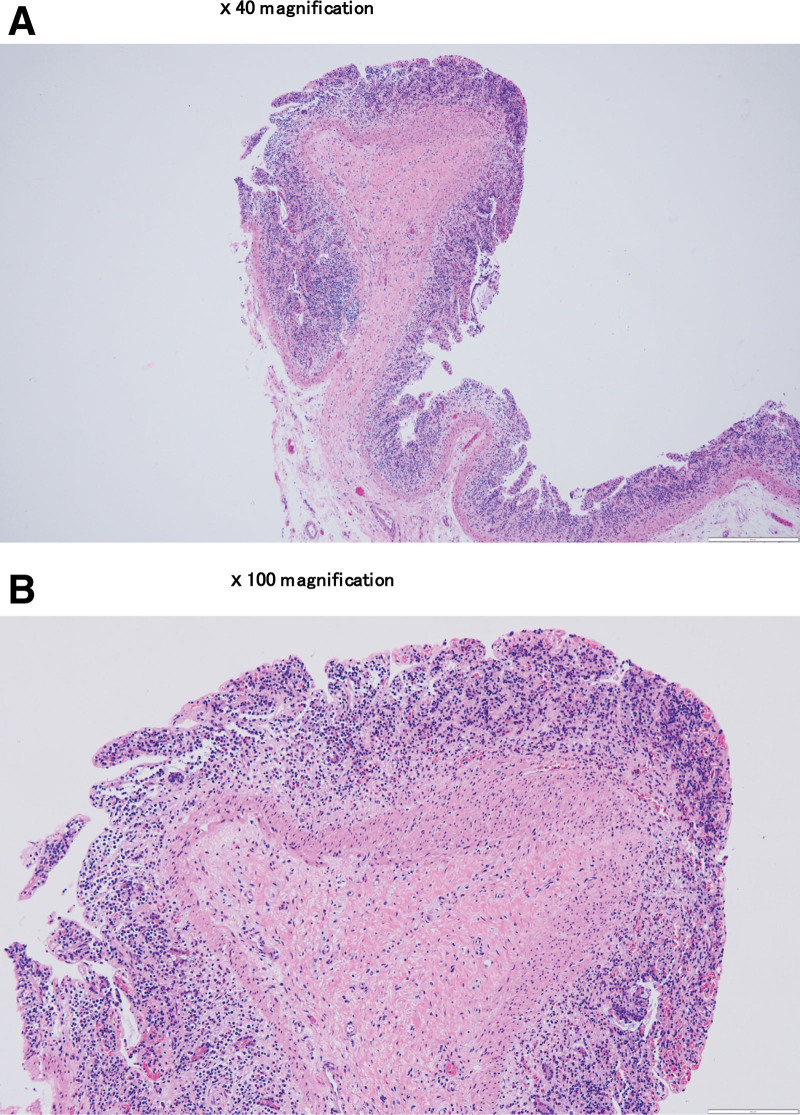
The pathologic findings. (A) ×40 magnification: mucosal infarction and prolapse. (B) ×100 magnification: muscularis mucosa thickening.

From the gross findings after surgery, we observed a diaphragm-like intraluminal obstruction lesion. There were 3–five palpable mass-like lesions in the resected small bowel (Fig. 3). Postoperatively, the patient complained of abdominal pain and constipation. Patient-controlled analgesics were administered, and additional painkillers such as pethidine were used. Laboratory findings recovered within the normal range within 4 days, but ileus on radiography persisted for a week during hospitalization. Pathological reports confirmed the presence of mucosal infarction and prolapse. The pathological assessment also indicated muscularis mucosa thickening (Fig. 4). On postoperative day (POD) 8, ileus and other symptoms improved, and he was discharged on POD 10. The entire process, from the first ER visit to the final follow-up, was organized into a timeline (Supplemental digital content, http://links.lww.com/MD/K700).

## 7. Discussion

SBDD is characterized by multiple lesions (3–70) and thin (2–4 mm) diaphragm-like septa, narrowing the bowel lumen to a pinhole-producing luminal occlusion. In previous studies of SBDD, multiple lesions were detected, and some segments were resected.^[7]^ However, in our case, palpable mass-like lesions were localized in 1 segment within 20 cm. We presumed that localized multiple lesions of SBDD may have been misinterpreted as simple small bowel obstruction due to an adhesion band.

Small bowel obstruction is usually caused by adhesions, hernias, malignancies, or inflammatory strictures.^[8]^ Moreover, a medical history of abdominal surgery is the main cause of adhesion and internal hernia. The patient, in this case report had mechanical obstruction of the small bowel without a history of operative treatment. As CT findings suggested small bowel obstruction due to an adhesion band, we overlooked the importance of the patient medication history. After SBDD was identified in the operating room, we rechecked the patient history of medications. In a review of medical records from local clinicians, we found that the patient had taken naproxen 500 mg 1 tab bid and polmacoxib 2 mg 1 cap as pro re nata for pain control due to spinal stenosis.

The pathophysiology of NSAID-induced small-bowel injuries is a 3-step hypothesis. First, NSAIDs inhibit mitochondrial oxidative phosphorylation of enterocytes. Second, inhibition of oxidative phosphorylation leads to the dysfunction of tight intracellular junctions; as a result, intestinal permeability is increased. Third, as intestinal permeability increases, enterocytes are exposed to bowel contents such as bile acids, hydrolytic and proteolytic enzymes, pancreatic secretions, and intestinal bacteria. This cascade results in neutrophil chemotaxis with activation of neutrophils, causing nonspecific inflammation and ulceration in the mucosa of the small bowel.^[4]^ This mucosal damage may lead to inflammation and ulceration, followed by repeated fibrosis and stricture formation. These local inflammatory changes are related to the disruption of membrane phospholipids in the bowel wall.^[9]^ Repeated mucosal injury from NSAIDs will cause the formation of circumferential strictures resembling a diaphragm. It has been proposed that the chronic cycle of injury and repair with the deposition of a collagenous scar results in intraluminal narrowing akin to a “drawstring.”^[10,11]^

This case report has limitations of the uncertain pathophysiology of SBDD, so we presume the usage of NSAIDs for over 3 years affected the occurrence of SBDD in this patient. Furthermore, we cannot explain why multiple lesions occur in a short segment (about 20 cm) of the small bowel.

In conclusion, we want to share the lessons learned from the experience with our patient with SBDD. First, a medical history of small bowel obstruction without an operative management history is essential; SBDD should be suspected when there is a history of long-term NSAID administration. Second, as SBDD can cause multiple lesions, surgeons should palpate the entire small bowel from the ligament of Treitz to the ileocecal valve to identify SBDD lesions. Third, as a remaining SBDD lesion can cause additional obstructive symptoms, clinicians should check the whole bowel using the squeezing technique to confirm that bowel contents pass smoothly in the small bowel. Finally, SBDD can be misdiagnosed as small bowel obstruction due to an adhesion band.

## Acknowledgments

Special thanks to illustrator M.K. Choi for helping us with the timeline.

## Author contributions

**Conceptualization:** Jae Hun Chung.

**Data curation:** Dong Won Lim.

**Formal analysis:** Si Hak Lee, Dong Hoon Shin.

**Project administration:** Jae Hun Chung.

**Supervision:** Sun-Hwi Hwang.

**Visualization:** Jae Hun Chung.

**Writing – original draft:** Youseok Jeong.

**Writing – review & editing:** Jae Hun Chung.

## Supplementary Material
