# Supplementary figures and images for: Small bowel diaphragm disease with multiple cluster lesions in one segment of the small bowel mimicking an adhesion band: A case report
Source: Medicine (Baltimore). 2023 Nov 24;102(47):e35235. doi: 10.1097/MD.0000000000035235 (PMC10681587; doi:10.1097/MD.0000000000035235)

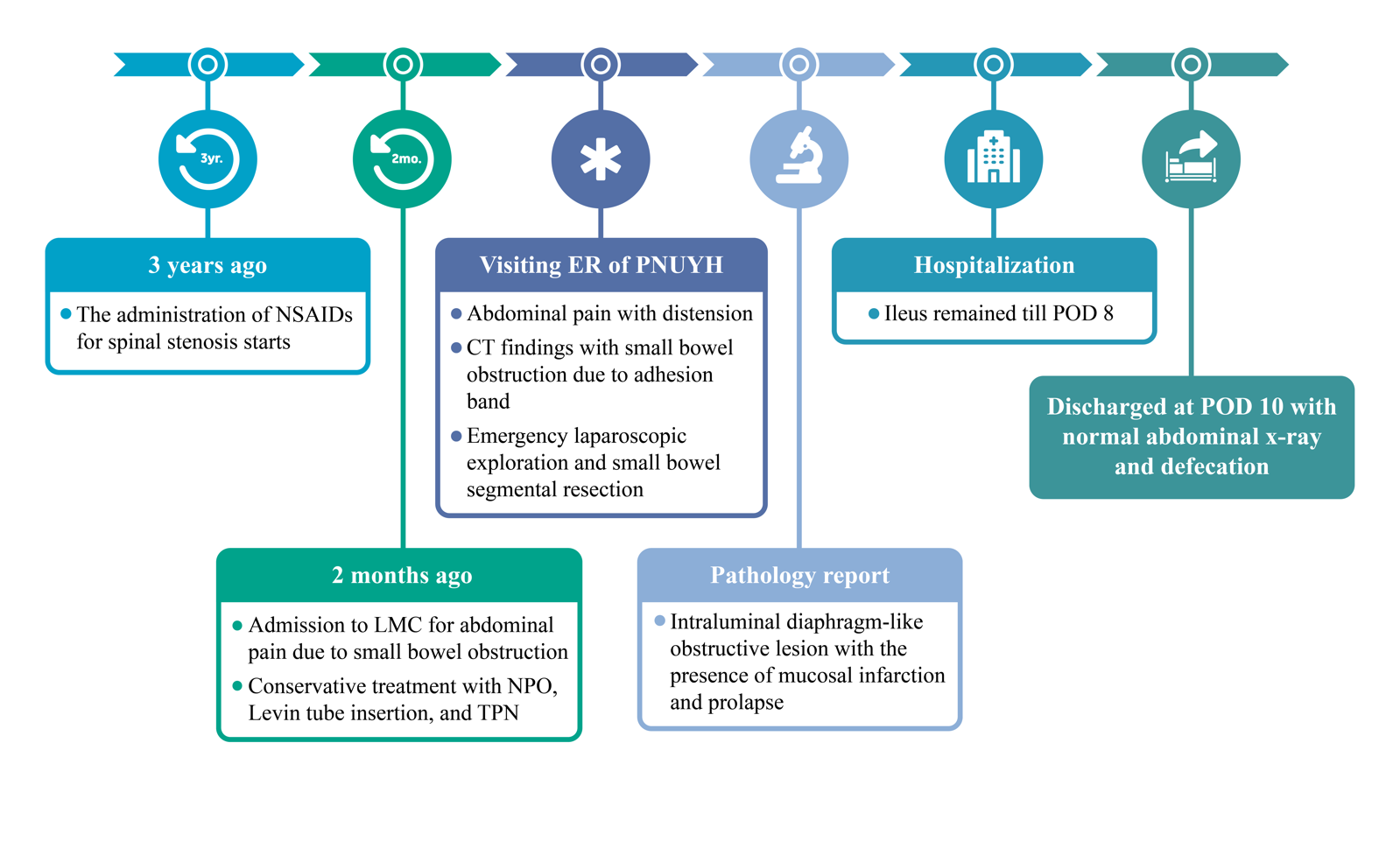

Supplement: Supplementary file 1 [file medi-102-e35235-s001.docx]
